# Cessation outcomes and healthcare provider advice to quit among tobacco users: A cross-sectional analysis of the 2018–2019 Tobacco Control Policy (TCP) India survey

**DOI:** 10.18332/tid/215706

**Published:** 2026-02-17

**Authors:** Prakash Babu Kodali, Mangesh S. Pednekar, Prakash C. Gupta, Anne C. K. Quah, Geoffrey Τ. Fong, Stella A. Bialous, Maya Vijayaraghavan

**Affiliations:** 1 Department of Public Health and Community Medicine, Central University of Kerala, Kasaragod, India; 2 Center for Tobacco Control Research and Education, University of California, San Francisco, United States; 3 Healis-Sekhsaria Institute for Public Health, Navi Mumbai, India; 4 Department of Psychology, University of Waterloo, Waterloo, Canada; 5 School of Public Health Sciences, University of Waterloo, Waterloo, Canada; 6 Ontario Institute for Cancer Research, Toronto, Canada; 7 Smoking Cessation Leadership Center, Division of General Internal Medicine, University of California, San Francisco, United States

**Keywords:** cessation services, quit attempts, successful quit, tobacco control, tobacco use cessation

## Abstract

**INTRODUCTION:**

Tobacco cessation is crucial to reducing morbidity and mortality in India. Through a secondary analysis of the Tobacco Control Policy (TCP) India Wave 3 (2018–2019) survey data, we examined cessation outcomes, including successful quitting, quit attempts, use of cessation services, and healthcare provider (HCP) advice to quit, among combustible, smokeless, and mixed (combustible and smokeless tobacco) users.

**METHODS:**

We conducted a cross-sectional analysis of the TCP India Wave 3 (2018–2019) survey data. The survey captured self-reported data on tobacco use and cessation using structured questionnaires. Participants included combustible tobacco (cigarette/bidi) users (n=977), smokeless tobacco users (n=5806), and mixed users (i.e. combustible and smokeless tobacco users, n=1157). Weighted prevalence estimates were calculated, and multivariable analysis evaluated factors associated with cessation outcomes.

**RESULTS:**

Successful quitting among lifetime tobacco users ranged from 6.3% to 12.4%. Among current users, past quit attempts ranged from 4.8% to 20.9%, and cessation services use in the latest quit attempt ranged from 5.8% to 9.3%. More combustible tobacco users (67.5%) reported receiving HCP advice to quit than smokeless tobacco users (40.5%). Combustible tobacco users aged ≥55 years (adjusted odds ratio, AOR=3.82; 95% CI: 2.06–7.07) reported higher odds of quitting compared to individuals aged 15–39 years. Smokeless tobacco users who reported that their ‘partner thinks a lot that they should quit tobacco use’ (AOR=2.21; 95% CI: 1.85–2.64) and who received HCP advice to quit (AOR=2.07; 95% CI: 1.65–2.59) had higher odds of attempting to quit than their respective counterparts. Mixed users who perceived tobacco ‘caused a lot of damage to their health’ had higher odds of receiving HCP advice to quit (AOR=2.47; 95% CI:1.16–5.29) compared to those reporting ‘not at all’.

**CONCLUSIONS:**

Cessation outcomes and HCP advice to quit are suboptimal across tobacco users. Longitudinal studies are needed to understand the role of anti-tobacco advertising campaigns and spousal support on cessation outcomes.

## INTRODUCTION

Globally 1.3 billion adults use tobacco products and India accounts for 20.5% of current global tobacco use burden^1-3^. India’s tobacco use landscape is characterized by combustible (cigarettes, and hand-rolled bidis), smokeless (chewing tobacco, ghutka, and khaini etc.) and mixed use (use of combustible and smokeless tobacco)^2^. Currently, >200 million Indian adults use smokeless tobacco products, and approximately 100 million use combustible tobacco^1,2^.

India’s tobacco control efforts include bans on advertisements and promotions, demand reduction strategies (such as anti-tobacco advertising campaigns), and provision of tobacco cessation services^1^. The anti-tobacco advertising campaigns include pictorial health warnings and health education campaigns through various media, including the internet, television, radio, cinema, and other mass media^1,4^. As a signatory to the World Health Organization (WHO) Framework Convention on Tobacco Control, India has demonstrated strong implementation of Article 14 by 1) mandating quit advice at routine healthcare visits; 2) making nicotine replacement therapy (NRT) and pharmacotherapy (bupropion and varenicline) available; and 3) providing cessation services through over 2000 tobacco cessation centers, government-sponsored mCessation (regular SMS-based support to quit tobacco use), and quitline services^1,5^.

Despite these interventions, cessation outcomes among people using tobacco in India remain sub-optimal. In 2016–2017, only 14.2% of combustible tobacco users and 6.5% of smokeless tobacco users successfully quit^6,7^. Further, 36.3% of combustible tobacco users, and 32% of smokeless tobacco users reported past one-year quit attempts, and only 13.4% and 10.9% of quit attempts, respectively, were supported by cessation services^6-8^. These cessation outcomes are low compared to other countries like Mexico, Brazil, and Russia, and have remained stagnant between 2009 and 2017^6,9-11^. Similar to the United States and European nations^12,13^, the healthcare provider (HCP) engagement in aiding cessation is limited, with only 51% of tobacco users who visited a HCP receiving advice to quit^6,14^.

Cessation outcomes are associated with sociodemographic characteristics, tobacco dependence, health status, awareness and attitudes, and social influences. Age, residence, education level, and employment are associated with past quit attempts and successful quitting^6,7^. Mixed users and individuals with tobacco dependence (i.e. using tobacco within the first 30 minutes of waking) are less likely to quit tobacco use or make quit attempts^14,15^. Awareness about the negative effects of tobacco use^7,15^ and poor health status^14,15^ are both associated with more attempts and higher quit rates. Social influences such as partners’ encouragement to quit tobacco use, exposure to anti-tobacco advertising, and restrictions on smoking at home have a positive impact on cessation behaviors^16^. Receiving advice to quit from HCP is also associated with quit attempts and successful quitting^6,7^. Older individuals, those with chronic conditions and combustible tobacco users are more likely to receive HCP quit advice during healthcare visits^14^.

Tobacco cessation outcomes, including successful quitting, quit attempts, cessation service use, and HCP advice to quit, reflect the successful implementation of broader tobacco control policies^1,7,11^. However, evidence comprehensively capturing these outcomes for both smoking and smokeless tobacco users in India is scarce. Existing studies assessed cessation behaviors independently among smoking or smokeless tobacco users^6-8,14,15^, and were primarily based on Global Adult Tobacco Surveys (GATS) and National Family Health Survey (NFHS; 2015–2016), which do not adequately capture the impact of tobacco control interventions such as quitline services, mCessation, and tobacco cessation centers that were expanded after 2016–2017^1,17^. While the Tobacco Control Project (TCP) India Wave 1 (2010–2011) and Wave 2 (2012–2013) surveys captured these data^18^, latest evidence comprehensively capturing cessation outcomes across various forms of tobacco use in India is lacking. Further, the influence of health status, partner support, tobacco use restrictions, and exposure to broader public health interventions, like anti-tobacco advertising campaigns, on cessation outcomes is less understood.

Using the latest TCP Wave 3 survey (2018–2019)^18^, we assessed the prevalence of four cessation outcomes: successful quitting, making quit attempts, cessation services use, and receiving HCP advice to quit among exclusive combustible tobacco, exclusive smokeless, and mixed users of both combustible and smokeless tobacco products in India and sought to explore factors associated with these cessation outcomes across these groups. We hypothesized that cessation outcomes would differ by the type of tobacco used, sociodemographic characteristics, health status, and social influences.

## METHODS

### Study design and study setting

We conducted a secondary analysis of cross-sectional data from the 2018–2019 TCP Wave 3 Survey. The TCP India Survey is part of the International Tobacco Control Policy Evaluation Project (ITC Project) surveys conducted across 31 countries to evaluate the impact of national-level tobacco control policies^18^. The TCP India Wave 3 Survey is the third round of the recontact and replenishment survey conducted in 2018–2019. Earlier rounds were conducted in 2010–2011 (Wave 1), and 2012–2013 (Wave 2). The TCP India Wave 3 surveyed a total of 10474 participants aged ≥15 years across four states: Bihar, Madhya Pradesh, Maharashtra, and West Bengal. It employed a multi-stage probability sampling approach and used standardized screeners, household and individual survey questionnaires^18^. The surveys were conducted in participants’ homes by trained field investigators through a manual and computer-assisted personal interviewing approach^18^.

### Study sample and operationalization

In this study, we analyzed a sample of lifetime tobacco users who were exclusive combustible tobacco users (n=977), exclusive smokeless tobacco users (n=5806), and mixed users (n=1157) surveyed at Wave 3. We defined lifetime combustible tobacco use as present or past exclusive use of combustible tobacco products (i.e. cigarettes and/or bidis) at least once a month or smoking 100 or more cigarettes and/or bidis in the lifetime^16,18^. We defined lifetime smokeless tobacco use as present or past exclusive use of smokeless tobacco products (e.g. gudhaku, gul, ghutka, khaini, mawa, mishri, paan masala with tobacco, plain chewing tobacco etc.) for at least once a month^16^. Lifetime mixed use was present or past use of both combustible and smokeless tobacco products^16,18^. We defined current users as those who use combustible and smokeless products daily or less than daily, whether exclusively or in combination. The HCP visits were defined as self-reported visits to a physician or any HCP, within six months before participating in the survey^14^.

### Outcomes

We defined successful quitting as self-reported status of having quit combustible and/or smokeless tobacco at the time of the survey in a lifetime user^11^. Past quit attempts were defined as any lifetime serious attempt to stop using combustible and/or smokeless tobacco products by a current user^9^. Cessation service use was defined as the use of approved cessation approaches (i.e. nicotine gum, nicotine patch, bupropion, quitline, counselling, mCessation, cessation clinic) during the latest attempt to quit tobacco use^6^. We defined HCP advice to quit as receipt of any advice to stop using tobacco products by a HCP within the last six months^14^. The outcomes were computed specific to the type of tobacco user, and their status of tobacco use. We provide details on how each variable was operationalized in Supplementary file 1.

### Covariates

*Sociodemographic variables*

The sociodemographic variables included: sex (female,male), residence (urban, rural), education level (no formal education, primary and middle school, secondary school, graduate or higher), and employment status (not employed, employed).

*Health status*

Health status was self-reported as: poor, average, good, and excellent.

*Perception that tobacco damaged health*

The perception that combustible and/or smokeless tobacco use damaged health was self-reported as: not at all, little damage, and a lot of damage.

*Tobacco use dependence*

We defined ‘tobacco use dependence’ as the use of combustible or smokeless tobacco products within 30 minutes of waking up^19,20^.

*Awareness about the health effects of tobacco*

We computed a composite score (range: 0–24; Cronbach’s alpha=0.93) for awareness about the health effects of smoking (cigarettes/bidi) as a cause for stroke, cancer, heart disease, and other conditions linked with tobacco use. Awareness of health effects of smokeless tobacco products (composite score: 0–6; Cronbach’s alpha=0.85) as cause for stroke, mouth cancer, throat cancer, heart disease, gum disease, and difficulty opening the mouth (Supplementary file 1).

*Social influences*

We defined partners’ perception of quitting tobacco use based on the responses to items ‘partner thinks you should quit tobacco (smoking/smokeless)’ with the responses ‘No/not applicable’, ‘yes somewhat’, and ‘yes a lot’.

Smoking restrictions at home were self-reported with responses: ‘allowed’, ‘allowed with restrictions’, and ‘not allowed’.

We defined the variable ‘anti-tobacco advertising motivated to quit’ (with responses ‘no/less likely to quit’ and ‘more likely to quit’) based on two items: 1) capturing participant’s exposure to anti-tobacco messages on media (Internet, Television, Cinema etc.); and 2) perception that exposure to anti-tobacco advertising has made them more or less likely to quit (Supplementary file 1).

### Analysis

The data cleaning and analysis was conducted using Statistical Package for Social Sciences (SPSS) version 27 [IBM Corp. IBM SPSS Statistics for Windows, Version 27.0. Armonk, NY: IBM Corp., 2020]. Rescaled cross-sectional weights for the TCP Wave 3 Survey data were applied to account for the complex survey design.

Descriptive statistics (i.e. weighted percentages) were calculated to report prevalence estimates of outcome variables. To examine the association of socioeconomic factors, health dimensions, and social influences with cessation outcomes of successful quitting, past quit attempts, and HCP advice to quit, we conducted binary logistic regression analysis for outcomes across combustible tobacco users and smokeless tobacco users, and multinomial logistic regression analysis for cessation outcomes among mixed users. We developed logistic regression models capturing the main effects and reported adjusted odds ratios (AORs) and 95% confidence intervals (CIs). We identified independent variables for the regression models based on prior literature on tobacco cessation^6-8,14-16^, and we considered socioeconomic factors as potential confounders. Due to only a few observations, we did not examine factors associated with ‘cessation service use’. We applied sampling weights, to adjust for complex sample design. We assessed multicollinearity using the variance inflation factor (VIF), and a VIF of <2.5 was considered acceptable in our analysis. We used a two-tailed significance level set at α≤0.05.

### Ethics approval and informed consent

The 2018–2019 TCP India wave 3 survey was approved by the office of research ethics, University of Waterloo, Canada (ORE#15722) and the Healis Sekhsaria Institute for Public Health International Research Board, India (IRB00007340). Informed consent was obtained from all survey participants. This study was a secondary analysis of de-identified data, which does not meet the definition of human subjects research and therefore did not require review by the Institutional Review Board at the University of California, San Francisco (UCSF).

## RESULTS

The majority of participants (n=5806; 73.1%) were smokeless tobacco users, followed by mixed users (use of combustible and smokeless tobacco) (n=1157; 14.6%), and combustible tobacco users (n=977; 12.3%). The majority of lifetime combustible tobacco users and mixed users were males (97.7%, 96.5%), while lifetime smokeless tobacco users had an equal distribution of females (47.2%) and males (52.8%) (Table 1).

**Table 1:** Cross-sectional analysis of characteristics of lifetime and current tobacco users surveyed in the 2018–2019 TCP India Survey (N=7940)

*Variables*	*Combustible tobacco users*		*SLT users*		*Mixed users*	
	*Lifetime users* *(N=977)*	*Current users* *(N=881)*	*Lifetime users* *(N=5806)*	*Current users* *(N=5125)*	*Lifetime users* *(N=1157)*	*Current users* *(N=604)*
**Age** (years)	**%**	**%**	**%**	**%**	**%**	**%**
15–39	29.9	31.1	38.6	38.2	33.4	42.6
40–54	34.8	35.9	33.1	33.5	37.4	37.6
≥55	35.2	33.0	28.3	28.2	29.2	19.8
**Sex**						
Female	2.3	2.4	47.2	45.3	3.5	5.0
Male	97.7	97.6	52.8	54.7	96.5	95.0
**Residence**						
Urban	69.2	68.9	73.3	74.3	77.8	78.1
Rural	30.8	31.1	26.7	25.7	22.2	21.9
**Education level**						
No formal education	18.1	13.9	26.7	26.7	16.0	19.2
Primary and middle school	35.3	37.1	33.1	34.0	41.5	42.4
Secondary school	29.6	24.3	29.8	29.8	31.1	27.2
Graduate or higher	17.0	24.8	10.3	9.5	11.3	11.2
**Employment status**						
Not employed	14.7	13.9	43.9	42.1	14.4	13.1
Employed	85.3	86.1	56.1	57.9	85.6	86.9
**Health status**						
Poor	5.0	4.9	3.6	3.4	5.5	5.9
Average	32.3	30.9	23.6	24.8	30.2	31.9
Good	51.9	53.5	47.4	48.0	49.5	50.9
Excellent	10.8	10.6	25.4	23.7	14.7	11.3
HCP visit*						
No	68.3	70.1	75.2	75.7	71.9	74.5
Yes	31.7	29.9	24.8	24.3	28.1	25.5
**Anti-tobacco advertising motivated to quit**						
No/less likely to quit	NA	83.5	NA	78.5	NA	80.0
More likely to quit		16.5		21.5		20.0
Awareness, mean (SD)^a^	20.2 (5.2)	20.0 (5.3)	4.7 (1.8)	4.7 (1.8)	NA	NA
**Perceived that tobacco use has damaged health**						
Not at all	40.8	41.8	63.0	64.0	54.0	49.6
Little damage	23.2	21.5	29.5	29.3	20.9	18.7
A lot of damage	35.9	36.7	7.5	6.8	25.0	31.6
**Minutes to first cigarette/bidi after waking**						
>30	NA	44.2	NA	NA	NA	64.4
≤30		55.8				35.6
**Partner thinks you should quit smoking**						
No/NA	NA	27.3			NA	32.5
Yes, somewhat		18.8	NA	NA		16.8
Yes, a lot		53.8				50.7
**Smoking at home**						
Allowed	40.5	43.1				
Allowed with restrictions	20.2	21.0	NA	NA	NA	NA
Not allowed	39.3	35.9				
**Minutes to smokeless tobacco use after waking**						
>30	NA	NA	NA	49.0	NA	60
≤30				51.0		40
**Partner thinks you should quit smokeless tobacco**						
No/NA			NA	48.5	NA	27.4
Yes, somewhat	NA	NA		16.6		19.8
Yes, a lot				34.8		52.7

%: weighted percentages. SLT: smokeless tobacco. HCP: healthcare provider. NA: not applicable.

a Awareness was computed as a composite index of 24 items capturing the awareness about health effects of cigarettes and bidis (composite score of 0–24) for combustible tobacco users; and six items (composite score of 0–6) for smokeless tobacco users.

* Includes HCP visit with or without advice to quit.

### Prevalence of successful quitting, quit attempts, and HCP advice to quit

**Figure 1:**
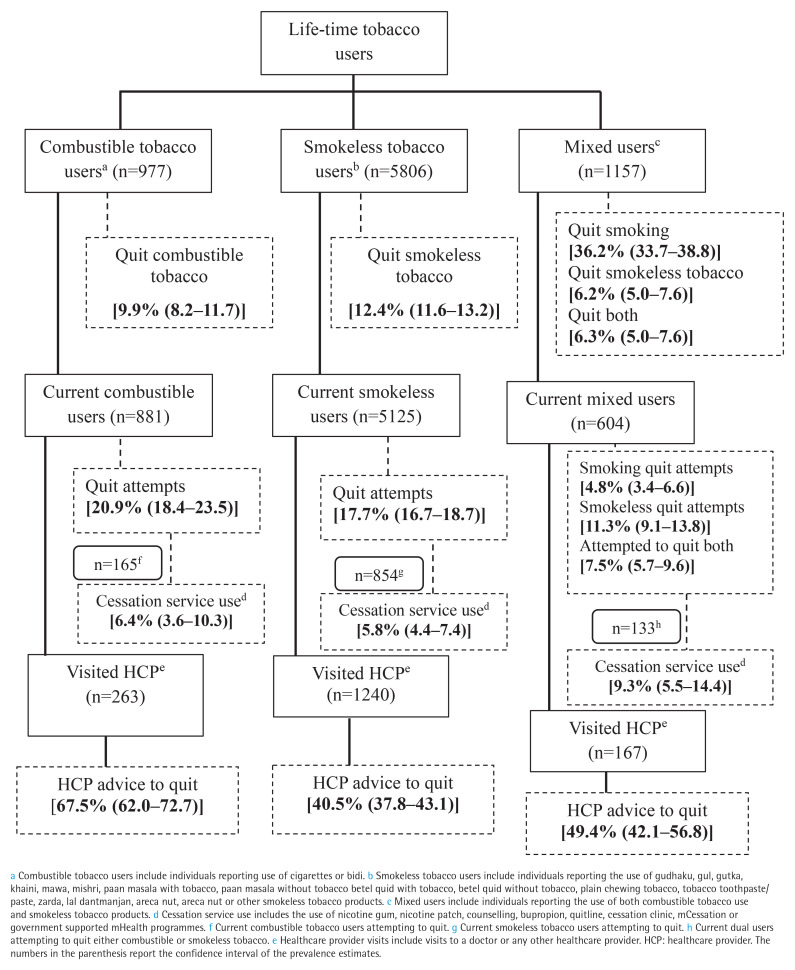
Prevalence of cessation outcomes and receipt of healthcare provider advice to quit among tobacco users, 2018–2019 TCP India Survey (N=7940)

The overall prevalence of successful quitting for all tobacco users was 12.4% (95% CI: 11.7–13.1). The prevalence of successful quitting among lifetime users of combustible tobacco (including combustible and mixed users) was 27.7% (95% CI: 25.9–29.5) and among mixed users was 6.3% (95% CI: 5.0–7.6). The prevalence of successful quitting among lifetime users of smokeless tobacco (including smokeless and mixed users) was 12.4% (95% CI: 11.7–13.2) (Figure 1).

The overall prevalence of quit attempts for current users of any form of tobacco products was 19.7% (95% CI: 18.9–20.6). The prevalence of past quit attempts was 20.9%, 17.7%, and 23.6% for current combustible tobacco users, smokeless tobacco users, and mixed users, respectively while the cessation service use among those attempting to quit was 6.4%, 5.8%, and 9.3% (Figure 1).

Among current users visiting an HCP, combustible tobacco users (n=263) had a higher proportion of receiving quit advice (67.5%) compared to smokeless tobacco users (n=1240) receiving advice to quit (40.5%) and mixed users (n=167) receiving advice to quit either of the tobacco products (49.6%) (Figure 1).

### Factors associated with successful quitting, quit attempts, and HCP advice to quit among combustible tobacco users

*Successful quitting*

Lifetime combustible tobacco users aged ≥55 years (AOR=3.82; 95% CI: 2.06–7.07) compared to individuals aged 15–39 years, individuals with higher awareness scores (AOR=1.14; 95% CI: 1.06–1.22) and living in homes where smoking was not allowed (AOR=5.58; 95% CI: 3.07–10.13) compared to participants reporting ‘smoking was allowed’ in their homes, were more likely to have quit smoking at the time compared to their counterparts (Table 2).

**Table 2:** Factors associated with cessation behaviors among participants who exclusively use combustible or smokeless tobacco during the 2018–2019 TCP India Survey

*Independent variables*	*Combustible tobacco ^a^*			*Smokeless tobacco ^b^*		
	*Successfully quit* *(N=977)*	*Quit attempts ^c^* *(N=881)*	*Healthcare provider advice to quit* *(N=263)*	*Successfully quit* *(N=5806)*	*Quit attempts ^c^* *(N=5125)*	*Healthcare provider advice to quit* *(N=1240)*
	*AOR (95% CI)*	*AOR (95% CI)*	*AOR (95% CI)*	*AOR (95% CI)*	*AOR (95% CI)*	*AOR (95% CI)*
**Age** (years)						
15–39 ®						
40–54	1.36 (0.71–2.61)	1.14 (0.72–1.82)	1.24 (0.53–2.87)	0.89 (0.73–1.08)	1.25 (1.04–1.50)*	0.93 (0.66–1.30)
≥55	3.82 (2.06–7.07)**	1.15 (0.70–1.87)	1.59 (0.71–3.58)	1.07 (0.86–1.33)	1.05 (0.85–1.29)	1.38 (0.97–1.95)
**Sex**						
Female ®						
Male	0.51 (0.11–2.43)	4.48 (0.50–40.41)	5.09 (0.53–48.89)	0.46 (0.37–0.58)**	0.66 (0.53–0.82)**	1.01 (0.73–1.40)
**Residence**						
Urban ®						
Rural	1.28 (0.75–2.18)	0.81 (0.52–1.26)	1.38 (0.64–2.97)	1.50 (1.26–1.80)**	0.48 (0.39–0.58)**	1.06 (0.78–1.43)
**Education level**						
No formal education ®						
Primary and middle school	1.18 (0.52–2.72)	1.91 (1.03–3.54)*	0.95 (0.38–2.33)	0.98 (0.78–1.24)	1.32 (1.07–1.64)*	0.46 (0.34–0.63)**
Secondary school	1.93 (0.83–4.49)	2.84 (1.48–5.47)**	0.51 (0.20–1.32)	1.42 (1.12–1.81)**	1.37 (1.09–1.73)**	0.41 (0.28–0.59)**
Graduate or higher	2.28 (0.93–5.60)	3.18 (1.54–6.56)**	0.91 (0.32–2.60)	2.59 (1.92–3.50)**	0.87 (0.61–1.23)	0.42 (0.24–0.74)**
**Employment status**						
Not employed ®						
Employed	0.64 (0.35–1.18)	0.87 (0.50–1.52)	0.94 (0.40–2.20)	0.81 (0.66–0.99)*	1.47 (1.19–1.81)**	1.21 (0.89–1.64)
**Tobacco use dependence^d^**						
No ®						
Yes	NA	1.50 (1.01–2.24)*	1.26 (0.66–2.43)	NA	1.21 (1.04–1.42)*	1.31 (1.02–1.69)*
**Perceived that tobacco use has damaged health^e^**						
Not at all ®						
Little damage	2.15 (1.24–3.73)**	2.90 (1.80–4.67)**	4.10 (1.56–10.79)**	1.33 (1.11–1.60)**	0.89 (0.75–1.06)	1.91 (1.45–2.52)**
A lot of damage	0.77 (0.44–1.37)	1.23 (0.79–1.90)	3.76 (1.84–7.70)**	2.35 (1.79–3.08)**	1.06 (0.79–1.42)	1.53 (0.98–2.40)
Awareness, mean (SD)^f^	1.14 (1.06–1.22)**	1.01 (0.97–1.05)	1.00 (0.94–1.06)	1.01 (0.97–1.07)	1.11 (1.06–1.17)	1.11 (1.04–1.19)
**Anti-tobacco advertising motivated to quit**						
No difference/less likely to quit ®						
More likely to quit	NA	2.13 (1.37–3.33)**	0.97 (0.44–2.12)	NA	1.82 (1.53–2.16)**	1.39 (1.04–1.86)*
**Partner thinks you should quit tobacco use^g^**						
No/NA ®						
Yes, somewhat	NA	0.66 (0.33–1.31)	0.56 (0.22–1.44)	NA	1.05 (0.82–1.34)	0.95 (0.65–1.39)
Yes a lot		1.97 (1.23–3.15)**	0.53 (0.24–1.20)		2.21 (1.85–2.64)**	1.77 (1.30–2.42)**
**Smoking at home**						
Allowed ®						
Allowed with restrictions	1.65 (0.78–3.51)	0.56 (0.32–0.99)*	2.17 (0.91–5.17)			
Not allowed	5.58 (3.07–10.12)**	2.19 (1.43–3.35)**	1.07 (0.53–2.17)	NA	NA	NA
**Health status**						
Poor ® Average	2.77 (0.92–8.35)	0.49 (0.22–1.11)	0.45 (0.13–1.53)	0.57 (0.37–0.88)*	0.75 (0.50–1.13)	0.74 (0.48–1.14)
Good	1.47 (0.49–4.45)	0.42 (0.19–0.94)*	0.56 (0.16–1.98)	0.85 (0.56–1.29)	0.58 (0.39–0.87)**	0.58 (0.37–0.90)*
Excellent	2.92 (0.84–5.60)	0.32 (1.54–6.56)*	1.27 (0.26–6.28)	1.40 (0.91–2.15)	0.48 (0.31–0.74)**	0.37 (0.21–0.66)**
**Received quit advice from healthcare provider**						
No/NA ®						
Yes	1.19 (0.69–2.05)	1.77 (1.12–2.79)*	NA	0.88 (0.65–1.19)	2.07 (1.65–2.59)**	NA

a Combustible tobacco users include individuals reporting use of cigarettes or bidi.

b Smokeless tobacco users include individuals reporting the use of gudhaku, gul, gutka, khaini, mawa, mishri, paan masala with tobacco, paan masala without tobacco betel quid with tobacco, betel quid without tobacco, plain chewing tobacco, tobacco toothpaste/paste, zarda, lal dantmanjan, areca nut or other smokeless tobacco products.

c Quit attempts are defined as any serious attempts to stop smoking by a tobacco user reporting the use of tobacco products at least less than once a month.

d Tobacco use dependence was defined as time to use smoking/smokeless tobacco products within 30 minutes of waking.

e Perception that combustible or smokeless tobacco products use has damaged health.

f Awareness was computed as a composite index of 24 items capturing the awareness about health effects of cigarettes and bidis (composite score of 0-24) for combustible tobacco users; and six items (composite score of 0-6) for smokeless tobacco users.

g Spouse/partner thinks that the respondent should quit smoking/smokeless tobacco products. The multivariable analysis was conducted employing binary logistic regression analysis. ® Reference categories. TCP: Tobacco Control Policy India Survey. AOR: adjusted odds ratio. NA: not applicable. The variables: 1) tobacco use dependence, 2) anti-tobacco advertising motivated to quit, and 3) partner thinks you should quit tobacco use were not measured among participants who successfully quit tobacco use as they were captured only among the current users; ‘Smoking at home’ was not included for the models capturing cessation outcomes across exclusive smokeless users.

* p<0.05.

** p<0.01.

*Quit attempts*

Among current users, individuals with education level of graduate or higher (AOR=3.18; 95% CI: 1.54–6.56) compared to those with no formal education, reporting ‘partner thinks they should quit smoking’ (AOR=1.97; 95% CI: 1.23–3.15) compared to those reporting no/not applicable, and receiving HCP advice to quit (AOR=1.77; 95% CI: 1.12–2.79) compared to those who did not receive it, had higher odds of quit attempts (Table 2).

*HCP advice to quit*

Among individuals visiting HCP, reporting tobacco use had caused ‘a lot of damage to health’ had greater odds of receiving advice to quit (AOR=3.76; 95% CI: 1.84–7.70) when compared to combustible tobacco users who reported that smoking has ‘not at all’ damaged their health (Table 2).

### Factors associated with successful quitting, quit attempts, and HCP advice to quit among smokeless tobacco users

*Successful quitting*

Males (AOR=0.46; 95% CI: 0.37–0.82) compared to females, and those reporting an average health status (AOR=0.57; 95% CI: 0.37–0.88) compared to individuals reporting a poor health status were less likely to quit smokeless tobacco (Table 2).

*Quit attempts*

Participants aged 40–54 years (AOR=1.25; 95% CI: 1.04–1.50) compared to those aged 15–39 years, reporting anti-tobacco advertising made them more-likely to quit (AOR=1.82; 95% CI: 1.53–2.16), and reporting receiving HCP advice to quit (AOR=2.07; 95% CI: 1.65–2.59) compared to not receiving one, were more likely to make quit attempts (Table 2).

*HCP advice to quit*

Among those visiting an HCP, individuals reporting excellent health (AOR=0.37; 95% CI: 0.21–0.66) compared to those reporting poor health, had lower odds of receiving advice to quit (Table 2).

### Factors associated with successful quitting, quit attempts, and HCP advice to quit among mixed users

*Successful quitting*

Mixed users aged ≥55 years had higher odds of quitting both smokeless and combustible tobacco (AOR=7.14; 95% CI: 3.33–15.31) compared to those in aged 15–39 years. A perception that tobacco use had caused ‘a lot of damage to health’ was associated with higher odds of quitting both products (AOR=3.90; 95% CI: 2.07–7.34) compared to those perceiving tobacco use has ‘not at all damaged health’ (Table 3).

**Table 3:** Factors associated with cessation behaviors among mixed users surveyed in the 2018–2019 TCP India Survey (N=1157)

*Independent variables*	*Lifetime mixed users*		
	*Successfully quit smokeless tobacco*	*Successfully quit combustible tobacco*	*Successfully quit both*
	*AOR (95% CI)*	*AOR (95% CI)*	*AOR (95% CI)*
**Age** (years)			
15–39 ®			
40–54	1.94 (1.02–3.68)*	1.89 (1.38–2.61)**	0.83 (1.36–5.90)**
≥55	4.90 (2.51–9.57)**	4.42 (3.05–6.40)**	7.14 (3.33–15.31)**
**Sex**			
Female ®			
Male	2.59 (0.32–21.01)	2.46 (1.07–5.63)*	1.45 (0.28–7.66)
**Residence**			
Urban ®			
Rural	1.01 (0.57–1.81)	0.89 (0.64–1.24)	1.95 (1.08–3.54)*
**Education level**			
No formal education ®			
Primary and middle school	1.69 (0.84–3.40)	1.50 (1.01–2.25)*	4.42 (1.44–13.57)**
Secondary school	1.18 (0.53–2.63)	2.36 (1.55–3.61)**	9.65 (3.12–29.91)**
Graduate or higher	1.76 (0.69–4.46)	1.52 (0.88–2.60)	8.76 (2.55–30.03)**
**Employment status**			
Not employed ®			
Employed	2.76 (1.08–7.06)*	0.95 (0.62–1.45)	0.57 (0.28–1.17)
**Perceived that tobacco has damaged health**			
Not at all ®			
Little damage	1.04 (0.53–2.03)	0.95 (0.69–1.32)	2.04 (0.93–4.46)
A lot of damage	1.18 (0.68–2.05)	0.16 (0.11–0.25)**	3.90 (2.07–7.34)**
**Health status**			
Poor ®			
Average	0.67 (0.25–1.78)	1.02 (0.53–1.95)	0.90 (0.24–3.30)
Good	0.81 (0.31–2.12)	1.03 (0.54–1.96)	1.93 (0.54–6.90)
Excellent	0.43 (0.11–1.63)	2.09 (1.03–4.24)*	4.95 (1.29–18.93)*
**Received healthcare provider advice to quit**			
No/NA ®			
Yes	1.59 (0.84–3.00)	1.18 (0.78–1.78)	1.22 (0.59–2.53)

The multivariable analysis was conducted employing multinomial logistic regression analysis. AOR: adjusted odds ratio. ® Reference categories. TCP: Tobacco Control Policy India Survey. HCP: healthcare provider.

* p<0.05,

** p<0.01.

*Quit attempts*

The mixed users reporting anti-tobacco advertisements motivated them to quit had quit combustible tobacco (AOR=4.49; 95% CI: 1.94–11.30), smokeless tobacco (AOR=2.20; 95% CI: 1.20–.04), and both combustible and smokeless tobacco (AOR=3.36; 95% CI: 1.66–6.78) compared to the mixed users reporting no difference/ less likely to quit (Table 4).

**Table 4:** Factors associated with quit attempts and healthcare provider advice to quit among mixed users surveyed in 2018–2019 TCP India Survey (N=604)

*Independent variables*	*Current mixed users* *(N=604) ^a^*		*Visited HCP* *(N=167) ^b^*	
	*Attempted to quit combustible tobacco use*	*Attempted to quit smokeless tobacco use*	*Attempted to quit both*	*Received HCP advice to quit*
	*AOR (95% CI)*	*AOR (95% CI)*	*AOR (95% CI)*	*AOR (95% CI)*
**Age** (years)				
15–39 ®				
40–54	1.06 (0.41–2.75)	0.55 (0.30–1.00)	1.05 (0.50–2.22)	0.74 (0.32–1.69)
≥55	1.25 (0.37–2.75)	0.59 (0.26–1.31)	2.13 (0.86–5.27)	0.88 (0.30–2.56)
**Sex**				
Female ®				
Male	2.00 (0.11–35.39)	0.27 (0.11–0.71)**	1.19 (0.14–10.00)	3.83 (0.42–35.07)
**Residence**				
Urban ®				
Rural	0.44 (0.13–1.49)	1.08 (0.57–2.04)	0.16 (0.04–0.61)**	1.08 (0.45–2.59)
**Education level**				
No formal education ®				
Primary and middle school	2.42 (0.63–9.23)	0.70 (0.34–1.43)	1.71 (0.62–4.69)	1.14 (0.45–2.87)
Secondary school	1.68 (0.40–7.14)	0.98 (0.46–2.08)	1.03 (0.33–3.18)	2.36 (0.82–6.81)
Graduate or higher	0.88 (0.12–6.40)	0.60 (0.20–1.86)	2.26 (0.69–7.38)	1.64 (0.37–7.23)
**Employment status**				
Not employed ®				
Employed	0.78 (0.21–2.89)	1.05 (0.45–2.45)	2.79 (0.83–9.37)	0.95 (0.32–2.83)
**Anti-tobacco advertising motivated to quit**				
No difference/less likely to quit ®				
More likely to quit	4.69 (1.94–11.30)**	2.20 (1.20–4.04)**	3.36 (1.66–6.78)**	0.85 (0.36–1.98)
**Health status**				
Poor ®				
Average	0.11 (0.06–2.12)	0.41 (0.14–1.25)	0.16 (0.05–0.52)**	1.81 (0.64–5.13)
Good	0.67 (0.16–2.88)	0.37 (0.12–1.11)	0.29 (0.09–0.92)*	0.76 (0.26–2.24)
Excellent	0.32 (0.07–1.47)	0.31 (0.08–1.20)	0.08 (0.01–0.48)**	0.68 (0.13–3.59)
**Received HCP advice to quit**				
No/NA ®				
Yes	1.56 (0.54–4.53)	2.02 (0.96–4.26)	2.00 (0.85–4.75)	NA
**Perceived that tobacco has damaged health**				
No damage ®				
Little damage	0.60 (0.10–3.50)	0.66 (0.32–1.37)	0.60 (0.20–1.76)	1.11 (0.36–3.42)
A lot of damage	4.23 (1.61–11.16)**	0.54 (0.28–1.07)	1.29 (0.63–2.64)	2.47 (1.16–5.29)*

a The multivariable analysis was conducted employing multinominal logistic regression analysis.

b The multivariable analysis was conducted employing binary logistic regression analysis. TCP: Tobacco Control Policy India Survey. AOR: adjusted odds ratio. HCP: healthcare provider. NA: not applicable. ® Reference categories.

* p<0.05.

** p <0.01.

*HCP advice to quit*

Mixed users reporting that tobacco use caused ‘a lot of damage to health’ were more likely to receive advice to quit from an HCP during the latest visit (AOR=2.47; 95% CI: 1.16–5.29) compared to those reporting no damage (Table 4).

## DISCUSSION

In this sample of tobacco users in India, individuals with a history of combustible tobacco use were more likely to quit, attempt to quit, use cessation services, and receive HCP advice to quit compared to smokeless tobacco users. Overall prevalence of quitting smoking among all combustible users was consistent with previous estimates^11^. The overall prevalence of quitting smokeless tobacco was higher than the smokeless tobacco quit prevalence of 8.7%–6.6% reported earlier^6,21^, and consistent with the declining trend of smokeless tobacco use in India^22,23^. Our study adds to the literature by providing quit rates disaggregated by tobacco use, and by showing that successful quitting is concentrated among mixed users.

Our estimates of quit attempts of 19.7% across all current tobacco users were lower than the 25% observed in the first wave of TCP survey (2010–2011) in India^16^. The decline is driven by the stagnation in quit attempts among combustible tobacco users (36.2% in 2009 and 36.4% in 2016), and smokeless tobacco users (33.7% in 2009 and 32% in 2016) observed previously^11,15^.

Consistent with previous studies, less than one in ten tobacco users used cessation services in their latest quit attempt^5,6,15^. The low use of cessation services could be due to: 1) fewer people attempting to quit, 2) lack of awareness, and 3) low density of available cessation support services^5,17^. Furthermore, the limited engagement of HCPs in providing quit advice cannot be ignored. Our estimates indicate combustible tobacco users were more likely to receive advice to quit^14,24^. while more than half of smokeless tobacco and mixed users are missed possibly due to lack of preparation of HCPs to intervene^24^, the social context of smokeless tobacco use, and perceived harmlessness of smokeless tobacco products^25,26^.

Previous studies reported that individuals aged ≥45 years are more likely to quit tobacco use^6,7^. Our estimates indicate a higher probability of quitting all forms of tobacco use in the older years (aged ≥55 years), contradicting the international evidence, which reports that older people find it harder to quit tobacco use^27^. In India, quit attempts are more prevalent among younger population, while older populations are more likely to successfully quit^6,7^. Although access to cessation support is equally limited, older individuals, owing to their health status, may be more inclined to attempt to quit, interact with a HCP, and receive advice to quit tobacco use from an HCP^6,7,14^. This also aligns with our findings, where individuals reporting that tobacco use caused ‘a little damage’ or ‘a lot of damage’ to their health were more likely to quit tobacco use compared to those reporting ‘no damage’. Our findings add to the evidence on the positive role of HCP advice to quit in improving quit attempts^6-8^.

Consistent with earlier studies, we found a higher probability of attempting to quit tobacco use among females, individuals with better education, and poor health status^14,16^. Among the mixed users, higher levels of education were associated with quitting smoking but not smokeless tobacco use^6^. Most mixed users reported quitting smoking but continued to use smokeless tobacco^15^. Similar to studies in the United States and China, we also found that partners’ support to quit tobacco use was associated with making quit attempts^28,29^ and combustible tobacco users living in homes where smoking is not allowed were more likely to quit smoking^30^. Qualitative studies in India have shown family is a powerful motivator to quit tobacco use^25,31^. However, further research is required to investigate the role of spousal support in tobacco cessation in the Indian context.

Anti-tobacco messaging campaigns that include messages on tobacco packages, public places, workplaces, and mass media have been shown to be associated with intention to quit and quit attempts^4,16^. However, <25% tobacco users perceived that the anti-tobacco advertising made them more likely to quit tobacco use, and not everyone was equally exposed to effective anti-tobacco campaigns^4^. This indicates the need to improve the quality, content, and acceptability of the anti-tobacco campaigns^4^, while simultaneously ensuring the provision of cessation services.

### Strengths and limitations

The study’s strength is its use of the latest wave of the TCP survey, and a comprehensive outlook to cessation outcomes across combustible, smokeless, and mixed users. The limitations include the use of self-reported items with a potential for recall, misclassification, and social desirability bias. Findings may not be generalizable beyond the four states where data collection took place or to other countries. We are unable to infer causal associations with a cross-sectional study design, and there may be a potential for residual unmeasured confounding even as we adjusted for known confounders. Despite these limitations, the study offers the latest estimates for tobacco use cessation in India, providing valuable insights to strengthen the country’s tobacco control efforts.

## CONCLUSIONS

In India, one in ten lifetime tobacco users successfully quit, while one in five current users attempted to quit, and less than one in ten tobacco quit attempts was supported by cessation services. The findings indicate the need to strengthen comprehensive tobacco control strategies and support cessation. Anti-tobacco advertising campaigns, tobacco use restrictions in home, spousal support and healthcare provider engagement could be crucial to improve cessation outcomes, and warrant further exploration in longitudinal cessation studies.

## Supplementary Material



## Data Availability

The data supporting this research are available from the following sources: In each country participating in the International Tobacco Control Policy Evaluation (ITC) Project, the data are jointly owned by the lead researcher(s) in that country and the ITC Project at the University of Waterloo. Data from the ITC Project are available to approved researchers 2 years after the date of issuance of cleaned data sets by the ITC Data Management Centre. Researchers interested in using ITC data are required to apply for approval by submitting an International Tobacco Control Data Repository (ITCDR) request application and subsequently to sign an ITCDR Data Usage Agreement. The criteria for data usage approval and the contents of the Data Usage Agreement are described online (http://www.itcproject.org). The authors of this article obtained the data following this application process. They did not have any special access privileges. Others would be able to access these data in the same manner as the authors.
