# Supplementary material for: Cessation outcomes and healthcare provider advice to quit among tobacco users: A cross-sectional analysis of the 2018–2019 Tobacco Control Policy (TCP) India survey
Source: Tob Induc Dis. 2026 Feb 17;24:10.18332/tid/215706. doi: 10.18332/tid/215706 (PMC12915482; doi:10.18332/tid/215706)
Supplement: Supplementary file 1 [file TID-24-24-s1.pdf]

Supplementary File 1

Dependent and Independent Variables, 2018-19 TCP India Survey

<table>
<tr><th colspan="2">Dependent Variables</th></tr>
<tr><td>Original variable/s</td><td>Recoded Variable</td></tr>
<tr><td>Past and Present Cigarette Use
Currently smoke cigarettes at least once a month
Currently smoke cigarettes less than once a month
Smoked cigarettes in the past but have since stopped
Have never smoked cigarettes
Refused/ Don’t know</td>
<td rowspan="3">Combustible tobacco use (Cigarette and Bidi)
0. Never used combustible tobacco (includes have never smoked cigarettes/bidi; refused/ don’t know)
1. Life time combustible tobacco use (includes currently smoke cigarettes/bidi at least once a month and less than once a month, and smoked cigarettes/bidi in past but since stopped)

Successful quitting of combustible tobacco products among life time combustible tobacco users
0. Successfully quit (includes Smoked Cigarette/Bidi in past but have quit)
1. Current user of combustible tobacco products

Smokeless tobacco use (Snuff, Gudhaku, Gul, Gutka, Khaini, Mawa, Mishri, Paan Masala with Tobacco, Paan Masala without Tobacco Betel Quid with tobacco, Betel Quid without tobacco, Chewing tobacco, Tobacco Tooth Paste, Zarda, Lal dant Manjan, Areca Nut)

0. Never used smokeless tobacco (have never used smokeless tobacco, refused, don’t know)
1. Life time smokeless tobacco use (includes currently use smokeless tobacco products/areca nut at least once a month and less than once a month, and used smokeless tobacco products/areca nut in past but since stopped)

Successful quitting of smokeless tobacco products

0. Successfully quit (Used smokeless products (areca nut or smokeless tobacco products) in past but since stopped)
1. Current user of smokeless tobacco products/arecanut

Successful quitting of both smoking and smokeless tobacco products

0. Successfully quit (Quit both smoking and smokeless tobacco)
1. Current user of both smoking and smokeless tobacco products</td></tr>
<tr><td>Past and Present Bidi use
Currently smoke bidis at least once a month
Currently smoke bidis less than once a month
Smoked bidis in the past but have since stopped
Have never smoked bidis
Refused/ Don’t know</td></tr>
<tr><td>How often do you use each of the following products (Snuff, Gudhaku, Gul, Gutka, Khaini, Mawa, Mishri, Paan Masala with Tobacco, Paan Masala without Tobacco Betel Quid with tobacco, Betel Quid without tobacco, Chewing tobacco, Tobacco Tooth Paste, Zarda, Lal dant Manjan, Areca Nut)

Currently use at least once a month
Currently use less than once a month
Used smokeless products (areca nut or smokeless tobacco) in the past but have since stopped
Have never used
Refused
Don't know</td></tr>
<tr><td>Have you ever made a serious attempt to stop using cigarettes
Yes
No
Refused
Don’t know
Not applicable

Have you ever made a serious attempt to stop using bidis
Yes
No
Refused
Don’t know
Not applicable

Have you ever made a serious attempt to stop using all smokeless tobacco products including areca nut</td>
<td>Attempted to quit combustible tobacco products (bidi and cigarette)
Yes
No (includes No/Refused/Don’t know/Not applicable)

Attempted to quit smokelesss tobacco products
Yes
No (includes No/Refused/Don’t know/Not applicable)

Attempted to quit both combustible and smokelesss tobacco products
Yes
No (includes No/Refused/Don’t know/Not applicable)</td></tr>
</table>

Yes<br>No<br>Refused<br>Don't know<br>Not applicable	
**Which of the following types of cessation help did you use as part of your most recent attempt to quit tobacco products (cigarette/bidi/smokeless tobacco/arecanut)?**<br><br>Nicotine gum<br>Nicotine patch<br>Bupropion<br>Quitline<br>Counselling<br>mCessation<br>Subscribe to government SMS cessation programme<br>Visited cessation clinic<br>Other<br>Refused<br>Don't know	**Used cessation services in the latest quit attempt**<br><br>0. Attempted quitting without cessation services (include others, refused, don't know)<br><br>1. Attempted quitting smoking/smokeless tobacco using cessation services. (includes nicotine gum, nicotine patch, bupropion, quitline, counselling, mCessation, subscription to gov. SMS cessation programme, and cessation clinic)
**Visited health professional**<br>Yes<br>No<br>Not applicable<br>Refused<br>Don't Know<br>**Advice from doctor to quit tobacco**<br>Yes<br>No<br>Not applicable<br>Refused<br>Don't Know	**Received healthcare provider quit advice among individuals visiting healthcare provider**<br><br>Yes<br>No (includes no, not applicable, don't know)
**Independent Variables**	
**Original variable/s**	**Recoded Variable**
**Age if the respondent at recruitment**<br><br>**Year of recruitment**	**Age group**<br>0. 15-39 years<br>**1.** 40-54 years<br>**2.** 55 years and above
**Highest level of education**<br>Illiterate<br>Little, no formal education<br>Up to primary school<br>Middle school<br>Secondary school<br>Graduate<br>Post graduate/professional degree<br>Above post graduate degree	**Education level**<br>0. Illiterate or no formal education<br>1. Primary and middle school<br>2. Secondary school<br>3. Graduate and above
**Occupation Group**<br>Professional, technical and related workers<br>Administrative, executive and managerial workers<br>Clerical and related workers<br>Sales workers<br>Service workers<br>Farmers, fisherman, hunters, loggers and related workers<br>Craft and related trades<br>Plant and machine operators<br>Elementary Occupations	**Occupational status**<br><br>0. Not employed (includes students, currently have no job, house wife)<br>1. Employed (includes rest of the occupation groups representing employment status)

Student<br>Currently have no job<br>Housewife<br>Others	
**Time to first smoke after waking**<br>5 min or less<br>6-30 mins<br>31-60 mins<br>More than 60 mins<br>Refused/Don't know	**Time to first smoke after waking**<br>0. >30 minutes (also includes refused/don't know)<br>1. Within 30 minutes
**Smoking cigarettes/bidi damaged your health**<br>Not at all<br>A little<br>A lot<br>Not applicable/Refused/don't know	**Perceived that smoking has damaged health**<br>0. Not at all (includes not at all, not applicable/refused/don't know)<br>1. Little damage<br>2. A lot of damage
**Time to first smokeless tobacco after waking**<br>5 minutes or less<br>6-30 minutes<br>31-60 minutes<br>More than 60 minutes<br>Refused<br>Don't know	**Time to smokeless tobacco after waking**<br>0. >30 minutes (also includes refused/don't know)<br>1. Within 30 minutes
**Smokeless tobacco damaged your health**<br>Not at all<br>A little<br>A lot<br>Refused<br>Don't know	**Perceived that smokeless tobacco has damaged health**<br>0. Not at all (includes not at all, not applicable/refused/don't know)<br>1. Little damage<br>2. A lot of damage
**Spouse thinks you should quit smoking**<br>Yes, a lot<br>Yes, somewhat<br>No<br>Not Applicable<br>Refused<br>Don't Know	**Spouse thinks you should quit smoking**<br>0. No (includes no/not applicable/ refused/ don't know)<br>1. Yes, somewhat<br>2. Yes a lot<br><br>**Spouse thinks that you should quit smokeless tobacco**<br>0. No (includes no/not applicable/ refused/ don't know)<br>1. Yes, somewhat<br>2. Yes a lot<br><br>**Spouse thinks that you should quit tobacco**<br>0. No (includes no/not applicable/ refused/ don't know)<br>1. Yes, somewhat<br>2. Yes a lot
**Spouse thinks you should quit smokeless tobacco**<br>Yes, a lot<br>Yes, somewhat<br>No<br>Not applicable<br>Refused<br>Don't Know	
**Anti-tobacco ads made you contemplate quitting tobacco**<br>More likely to quit using tobacco<br>Less likely to quit using tobacco<br>Made no difference<br>Refused<br>Don't know	**Anti-tobacco ads made you contemplate quitting**<br>0. No difference (includes made no difference, refused, don't know)<br>1. Less likely to quit<br>2. More likely to quit<br><br>**Anti-tobacco advertising motivated to quit**<br>0. No difference/less likely to quit<br>1. More likely to quit
**Smoking cigarette causes (Individual items with the responses-**Yes; No; Not Applicable; Refused; Don't Know**)**<br><br>Stroke<br>Impotence<br>Mouth Cancer<br>Throat Cancer<br>Lung Cancer	**Awareness about health effects of smoking**<br><br>Each item was converted to binary responses with Yes recoded as "1" and rest recoded as "0".<br><br>Sum of all 24 items was done to create a composite score ranging from 0-24

Heart disease<br>Tuberculosis<br>Harm to unborn<br>Second hand smoke causes lung cancer<br>Second hand smoke heart disease<br>Second hand smoke asthma<br>Second hand smoke harms unborn	
**Smoking bidi causes (Individual items with the responses-**Yes; No; Not Applicable; Refused; Don't Know**)**<br><br>Stroke<br>Impotence<br>Mouth Cancer<br>Throat Cancer<br>Lung Cancer<br>Heart disease<br>Tuberculosis<br>Harm to unborn<br>Second hand smoke causes lung cancer<br>Second hand smoke heart disease<br>Second hand smoke asthma<br>Second hand smoke harms unborn	
**Smokeless tobacco causes (Individual items with the responses-**Yes; No; Not Applicable; Refused; Don't Know)<br><br>Stroke in users<br>Mouth cancer in users<br>Throat cancer in users<br>Hear diseases in users<br>Gum diseases in users<br>Difficulty to open mouth in users	**Composite score of Awareness about health effects of smokeless tobacco**<br><br>Each item was converted to binary responses with Yes recoded as "1" and rest recoded as "0".<br><br>Sum of all six item was done to create a composite score ranging from 0-6
**Smoking at home**<br>Smoking is not allowed in any indoor room inside home<br>Smoking is allowed only in some rooms inside home<br>No rules or restrictions<br>Not applicable/Refused/don't know	**Smoking at home**<br>0. Not allowed<br>1. Allowed in some rooms inside homes<br>2. No rules or restrictions (also include Not applicable/Refused/don't know)
**Health Status**<br>Poor<br>Average<br>Good<br>Excellent<br>Not applicable/Refused/don't know	**Health Status**<br>0. Poor (also include not-applicable/refused/don't know)<br>1. Average<br>2. Good<br>3. Excellent

Supplementary file 2

Table 1: Unadjusted odds ratios reporting the factors associated with cessation behaviours among participants who exclusively use combustible or smokeless tobacco during the 2018-19 TCP India Survey.

**Independent variables**	**Combustible tobacco [a]**			**Smokeless tobacco [b]**		
	**Successfully Quit (n=977)**	**Quit Attempts [c] (n=881)**	**Healthcare provider advice to quit (n=263)**	**Successfully Quit (n=5806)**	**Quit Attempts [c] (n=5125)**	**Healthcare provider advice to quit (n=1240)**
	**AOR (95% CI)**	**AOR (95% CI)**	**AOR (95% CI)**	**AOR (95% CI)**	**AOR (95% CI)**	**AOR (95% CI)**
**Age group**						
15-39 (ref)						
40-54	2.57 (1.51-4.39)*	1.21 (0.80-1.81)	0.92 (0.45-1.88)	1.03 (0.86-1.24)	1.02 (0.85-1.24)	0.72 (0.53-0.97)
≥55	1.17 (0.64-2.12)	1.34 (0.89-2.02)	0.79 (0.40-1.56)	1.50 (1.27-1.77)**	1.47 (1.24-1.74)**	0.95 (0.69-1.31)
**Sex**						
Female (ref)						
Male	1.00 (0.26-3.87)	5.83 (0.73-46.35)	0.27 (0.04-1.72)	1.04 (0.90-1.20)	0.95 (0.83-1.10)	1.00 (0.79-1.25)
**Place of residence**						
Urban (ref)						
Rural	1.03 (0.67-1.61)	0.72 (0.50-1.04)	0.84 (0.45-1.55)	0.46 (0.38-0.55)**	0.47 (0.39-0.58)**	0.88 (0.67-1.17)
**Education level**						
No formal education (ref)						
Primary and middle school	2.71 (1.28-5.77)**	2.33 (1.33-4.06)*	0.94 (0.44-1.97)	1.53 (1.26-1.85)**	1.51 (1.24-1.83)**	2.06 (1.25-3.39)**
Secondary school	2.34 (1.15-4.74)*	2.79 (1.58-4.91)**	1.30 (0.59-2.85)	1.55 (1.28-1.89)**	1.59 (1.30-1.93)**	1.89 (1.40-2.55)**
Graduate and higher	1.51 (0.73-3.12)	2.90 (1.56-5.37)**	0.76 (0.32-1.82)	0.76 (0.56-1.04)	0.84 (0.61-1.15)	1.70 (1.29-2.24)**
**Occupational status**						
Not employed (ref)						
Employed	0.59 (0.36-0.99)*	0.95 (0.60-1.52)	0.91 (0.47-1.79)	1.49 (1.29-1.73)**	1.39 (1.20-1.62)**	0.89 (0.71-1.12)
**Tobacco use dependence [α]**						
No (ref)						
Yes	NA	1.05 (0.76-1.45)	1.27 (0.74-2.17)	NA	1.54 (1.33-1.78)**	0.73 (0.58-0.92)**
**Perceived that tobacco use has damaged health [β]**						
Not at all (ref)						
Little damage	2.45 (1.51-3.97)**	2.44 (1.62-3.67)**	1.21 (0.54-2.71)	0.95 (0.81-1.11)	0.96 (0.82-1.13)	0.54 (0.42-0.70)**
A lot of damage	0.88 (0.52-1.49)	1.17 (0.79-1.72)	3.96 (1.77-8.86)**	1.09 (0.83-1.42)	1.26 (0.96-1.66)	0.52 (0.34-0.79)**

**Awareness (Mean, SD)** [γ]	1.13 (1.06-1.21)**	1.02 (0.99-1.05)	0.98 (0.93-1.03)	1.17 (1.12-1.23)**	1.18 (1.13-1.24)**	0.91 (0.86-0.97)**
**Anti-tobacco advertising motivated to quit**						
No difference/Less likely to quit (ref)						
More likely to quit	NA	2.15 (1.45-3.18)**	1.04 (0.52-2.02)	NA	2.34 (1.99-2.73)**	0.77 (0.59-1.00)
**Partner thinks you should quit tobacco use** [δ]						
No/Not applicable (ref)						
Yes, somewhat	NA	0.58 (0.32-1.03)	1.12 (0.51-2.47)	NA	1.02 (0.81-1.29)	1.28 (0.91-1.79)
Yes a lot		1.72 (1.17-2.54)**	1.37 (0.69-2.73)		2.56 (2.19-3.00)**	0.71 (0.55-0.91)**
**Smoking at home**						
Allowed (ref)						
Allowed with restrictions	2.00 (0.99-4.06)	0.72 (0.43-1.20)	0.40 (0.19-0.86)*			
Not allowed	5.00 (2.88-8.68)**	2.27 (1.59-3.24)**	1.09 (0.61-1.96)	NA	NA	NA
**Health status**						
Poor (ref)						
Average	0.95 (0.35-2.63)	0.33 (0.17-0.65)**	2.94 (0.94-9.17)	0.47 (0.33-0.68)**	0.84 (0.58-1.21)	1.42 (0.95-2.11)
Good	0.57 (0.23-1.40)	0.30 (0.16-0.57)**	3.48 (1.12-10.80)*	0.67 (0.47-0.95)*	0.60 (0.42-0.86)**	1.80 (1.21-2.69)**
Excellent	1.07 (0.44-2.61)	0.30 (1.14-0.67)**	2.34 (0.58-9.45)	0.97 (0.67-1.38)	0.46 (0.32-0.68)**	3.07 (1.83-5.17)**
**Received quit advice from healthcare provider**						
No/NA (ref)						
Yes	1.48 (0.93-2.35)	1.84 (1.27-2.66)**	NA	2.49 (2.06-3.04)**	2.49 (2.03-3.04)**	NA

[a] Combustible tobacco users include individuals reporting use of cigarettes or bidi

[b] Smokeless tobacco users include individuals reporting the use of gudhaku, gul, gutka, khaini, mawa, mishri, paan masala with tobacco, paan masala without tobacco betel quid with tobacco, betel quid without tobacco, plain chewing tobacco, tobacco toothpaste/paste, zarda, lal dantmanjan, areca nut or other smokeless tobacco products.

[c] Quit attempts are defined as any serious attempts to stop smoking by a tobacco user reporting the use of tobacco products at least less than once a month

[α] Tobacco use dependence was defined as time to use smoking/smokeless tobacco products within 30 minutes of waking.

[β] Perception that combustible or smokeless tobacco products use has damaged health.

[γ] Awareness was computed as a composite index of 24 items capturing the awareness about health effects of cigarettes and bidis (composite score of 0-24) for combustible tobacco users; and six items (composite score of 0-6) for smokeless tobacco users.

[δ] Spouse/partner thinks that the respondent should quit smoking/smokeless tobacco products.

Note: The multivariable analysis was conducted employing binary logistic regression analysis *p-value < 0.05, **p-value < 0.01; ref= Reference category; TCP= Tobacco Control Policy India Survey; AOR= Adjusted Odds Ratio; CI= Confidence Interval; SD= Standard Deviation; NA= Not applicable. The variables a) Tobacco use dependence, b) anti-tobacco advertising motivated to quit, and c) partner thinks you should quit tobacco use were not measured among participants who successfully quit tobacco use as they were captured only among the current users; 'Smoking at home' was not included for the models capturing cessation outcomes across exclusive smokeless users.

Table 2: Unadjusted odds ratios reporting factors associated with cessation behaviours among mixed users surveyed in the 2018-19 TCP India Survey (n=1157)

**Independent variables**	**Lifetime mixed users**		
	**Successfully Quit smokeless tobacco AOR (95% CI)**	**Successfully Quit combustible tobacco AOR (95% CI)**	**Successfully Quit both AOR (95% CI)**
**Age group**			
15-39 (ref)			
40-54	2.05 (1.09-3.86)*	1.63 (1.21-2.19)**	2.58 (1.28-5.19)**
≥55	4.21 (2.23-7.94)**	3.12 (2.26-4.30)**	6.03 (3.02-12.06)**
**Sex**			
Female (ref)			
Male	4.13 (0.54-31.66)	2.51 (1.18-5.33)*	2.09 (0.45-9.65)
**Place of residence**			
Urban (ref)			
Rural	1.10 (0.63-1.92)	0.94 (0.70-1.27)	1.64 (0.97-2.79)
**Education level**			
No formal education (ref)			
Primary and middle school	1.40 (0.71-2.76)	1.36 (0.94-1.96)	2.75 (0.95-7.96)
Secondary school	0.96 (0.44-2.07)	1.92 (1.31-2.81)**	5.18 (1.81-14.88)**
Graduate and higher	1.33 (0.54-3.24)	1.29 (0.79-2.11)	5.60 (1.79-17.52)**
**Occupational status**			
Not employed (ref)			
Employed	1.73 (0.74-4.06)	0.81 (0.57-1.15)	0.47 (0.26-0.85)
**Perceived that tobacco has damaged health**			
Not at all (ref)			
Little damage	1.16 (0.61-2.24)	0.98 (0.73-1.34)	2.06 (0.97-4.40)
A lot of damage	1.41 (0.83-2.39)	0.19 (0.13-0.28)**	4.12 (2.28-7.45)**
**Health status**			
Poor (ref)			
Average	0.72 (0.28-1.85)	1.14 (0.63-2.05)	0.83 (0.2502.71)
Good	0.76 (0.31-1.87)	1.17 (0.66-2.07)	1.11 (0.36-3.42)
Excellent	0.35 (0.10-1.28)	2.16 (1.15-4.06)*	2.50 (0.76-8.26)
**Received healthcare provider advice to quit**			
No/NA (ref)			
Yes	1.74 (0.95-3.16)	1.01 (0.70-1.45)	1.50 (0.79-2.84)

Note: The multivariable analysis was conducted employing multinomial logistic regression analysis *p-value < 0.05, **p-value < 0.01; ref= Reference category; TCP= Tobacco Control Policy India Survey; HCP= Healthcare provider; AOR= Adjusted Odds Ratio; CI= Confidence Interval

Table 3: Unadjusted odds ratio reporting factors associated with quit attempts and healthcare provider advice to quit among mixed users surveyed in 2018-19 TCP India Survey (n=604)

**Independent variables**	**Current Mixed users (n=604)** [a]			**Visited HCP (n=167)** [b]
	**Attempted to quit combustible tobacco use AOR (95% CI)**	**Attempted to quit smokeless tobacco use AOR (95% CI)**	**Attempted to quit both AOR (95% CI)**	**Received HCP advice to quit AOR (95% CI)**
**Age group**				
15-39 (ref)				
40-54	1.37 (0.56-3.33)	0.59 (0.33-1.05)	1.15 (0.57-2.32)	0.84 (0.40-1.77)
≥55	1.44 (0.50-4.12)	0.63 (0.31-1.29)	1.63 (0.75-3.56)	0.97 (0.41-2.29)
**Sex**				
Female (ref)				
Male	2.13 (0.14-32.75)	0.29 (0.13-0.67)**	1.19 (0.26-13.86)	2.76 (0.34-22.45)
**Place of residence**				
Urban (ref)				
Rural	0.53 (0.18-1.61)	0.99 (0.55-1.80)	0.19 (0.05-0.69)*	0.75 (0.35-1.64)
**Education level**				
No formal education (ref)				
Primary and middle school	2.12 (0.60-7.48)	0.79 (0.40-1.57)	1.62 (0.63-4.13)	0.94 (0.40-2.16)
Secondary school	2.00 (0.52-7.62)	1.14 (0.57-2.30)	1.24 (0.44-3.52)	1.85 (0.72-4.75)
Graduate and higher	0.99 (0.15-6.57)	0.57 (0.19-1.66)	2.56 (0.86-7.60)	1.50 (0.38-5.91)
**Occupational status**				
Not employed (ref)				
Employed	0.80 (0.27-2.35)	0.81 (0.40-1.63)	1.41 (0.51-3.91)	1.03 (0.45-2.39)
**Anti-tobacco advertising motivated to quit**				
No difference/ Less likely to quit (ref)				
More likely to quit	3.98 (1.78-8.90)**	2.17 (1.22-3.86)**	3.12 (1.63-5.97)**	0.68 (0.32-1.43)
**Health status**				
Poor (ref)				
Average	0.28 (0.07-1.10)	0.58 (0.21-1.64)	0.23 (0.08-0.68)**	1.46 (0.58-3.69)
Good	0.38 (0.11-1.32)	0.49 (0.18-1.34)	0.33 (0.13-0.87)*	0.85 (0.33-2.21)
Excellent	0.06 (0.01-0.95)*	0.43 (0.13-1.46)	0.11 (0.02-0.55)**	0.71 (0.16-3.24)
**Received HCP advice to quit**				
No/NA (ref)				
Yes	2.27 (0.86-5.95)	1.72 (0.86-3.43)	2.45 (1.16-5.17)*	NA
**Perceived that tobacco has damaged health**				
No damage (ref)				
Little damage	0.55 (0.10-3.11)	0.73 (0.37-1.43)	0.53 (0.19-1.47)	1.20 (0.44-3.28)
A Lot of damage	4.26 (1.74-10.41)**	0.60 (0.32-1.13)	1.51 (0.79-2.87)	2.20 (1.10-4.39)*

a= The multivariable analysis was conducted employing multinominal logistic regression analysis
b= The multivariable analysis was conducted employing binary logistic regression analysis
TCP= Tobacco Control Policy India Survey; AOR= Adjusted Odds Ratio; CI= Confidence Interval; HCP= Healthcare provider; ref= Reference Category; NA= Not applicable; *p value <0.05; **p-value <0.01.

© 2026 Kodali P.B. et al.
